# Prophylactic Antibiotic Use and Clinical Outcomes in Patients With Child–Pugh A Cirrhosis and Acute Variceal Bleeding: A Retrospective Cohort Study

**DOI:** 10.1155/cjgh/2030627

**Published:** 2026-04-13

**Authors:** Jiajia He, Ju Huang, Guofeng Liu, Yuping He, Xiaoze Wang, Jinlin Yang, Li Yang, Xiao Li, Xuefeng Luo

**Affiliations:** ^1^ From the Department of Gastroenterology and Hepatology, Sichuan University–University of Oxford Huaxi Joint Centre for Gastrointestinal Cancer, West China Hospital, Sichuan University, Chengdu, China, scu.edu.cn; ^2^ From the Department of Interventional Therapy, National Cancer Center/National Clinical Research Center for Cancer/Cancer Hospital, Chinese Academy of Medical Sciences and Peking Union Medical College, 17 Panjiayuan Nanli Chaoyang District, Beijing, 100021, China, cacms.ac.cn

## Abstract

**Background & Aims:**

Prophylactic use of antibiotics was found to decrease mortality in patients with cirrhosis and acute variceal bleeding (AVB). Patients with Child A cirrhosis have a low risk of treatment failure and mortality. The present study was designed to investigate the association between antibiotics and Child–Pugh A patients with AVB.

**Methods:**

This retrospective analysis was conducted on Child–Pugh class A cirrhotic patients presenting with AVB at West China Hospital between January 2016 and November 2022. The outcome indicators evaluated in this study include the rate of treatment failure within five days, mortality within 6 weeks, incidence of nosocomial infections, and 1‐year cumulative mortality.

**Results:**

This study ultimately enrolled 237 patients. The day of patients’ admission was defined as Day 0. A total of 117 patients received prophylactic use of antibiotics on Days 0–1 (Group A), and 120 patients did not receive antibiotics on Days 0–1 (Group B). Baseline characteristics were well‐balanced between the two groups. The 5‐day treatment failure was 8.5% in Group A and 6.7% in Group B (*p* = 0.63). The 6‐week mortality was 1.71% in Group A and 0% in Group B (*p* = 0.24). The incidence of nosocomial infection (5.98% vs. 6.67%, *p* = 1.00) and 1‐year cumulative mortality (4.81% vs. 1.89%, *p* = 0.40) was comparable between the two groups.

**Conclusions:**

The antibiotics’ prophylactic use was not associated with 5‐day treatment failure or 6‐week mortality. Prophylactic use of antibiotics may not be routinely necessary in patients with Child A cirrhosis and AVB.

## 1. Introduction

Acute variceal bleeding (AVB) is a major decompensation event of cirrhosis and is associated with high mortality, with rates reaching 20% within 6 weeks [1, 2]. Previous studies have established a strong link between bacterial infection and poorer outcomes in these patients, including higher rates of treatment failure and mortality [3, 4]. The risk of mortality in infected patients can be up to four times greater than in non‐infected individuals [5]. Evidence from multiple randomized controlled trials indicates that prophylactic antibiotic use significantly lowers infection rates, reduces the risk of failed hemostasis [6–8], and improves overall survival [9–14]. Recent findings also showed benefits even in patients with acute‐on‐chronic liver failure (ACLF) [15]. As a result, antibiotic prophylaxis is now a guideline‐recommended measure for cirrhotic patients presenting with AVB [16–19].

However, in patients with Child–Pugh class A cirrhosis, baseline infection risk and 6‐week mortality are relatively low [20]. Some studies suggest that prophylactic antibiotics do not significantly influence clinical outcomes in this subgroup [21–24]. Consequently, these findings suggest that prophylactic antibiotic administration may not be consistently necessary [25]. At the same time, drug resistance or other side effects caused by antibiotics should also be considered [26, 27]. The value of prophylactic antibiotics in Child–Pugh A patients with AVB has yet to be established [11], prompting this retrospective analysis. This study aims to assess the association between prophylactic antibiotics and the outcomes of Child–Pugh A patients with AVB.

## 2. Materials and Methods

Between January 2016 and November 2022, 1576 patients with cirrhosis and AVB admitted to West China Hospital were included in this retrospective analysis. The day of patients’ admission was defined as Day 0. A total of 877 patients with Child B cirrhosis and 357 patients with Child C were excluded. Additionally, 74 patients were excluded from the study due to co‐existing infections or infection at Days 0–1 (*n* = 43) and advanced tumors (*n* = 31). Patients who were given prophylactic antibiotics after Day 1 were also excluded (*n* = 31). The treatment regimen for AVB included vasoactive drugs, endoscopic treatments, and/or interventional therapies. The use of prophylactic antibiotics was determined by the treating physicians. Patients were categorized as Group A if antibiotics were initiated on Day 0 or Day 1 in the absence of infection, and others were Group B if no antibiotics were given on Days 0–1.

Infection investigations were conducted for all patients upon admission. Infections were diagnosed based on the following criteria: (1) Spontaneous bacterial peritonitis: an ascites polymorphonuclear cell count of ≥ 250 cells/mm^3^, while Grade A patients rarely have ascites, and puncture is not routinely performed. Therefore, if the patient was accompanied by abdominal pain, tenderness, rebound tenderness, or muscle stiffness, then they were excluded from the study. (2) Pulmonary infection: characterized by new or progressive infiltrates, consolidation, or cavitation on chest x‐ray, along with clinical signs of infection such as fever exceeding 38°C, cough, sputum production, dyspnea, and other symptoms; (3) Urinary tract infection: identified by symptoms such as frequent urination, urgency, dysuria, a urinary leukocyte count exceeding 15 cells per high‐power field, and a positive urine culture; and (4) Bacteremia: defined by a fever with a body temperature higher than 38°C and positive blood cultures. Since patients were all Grade A and the incidence of infection was relatively low, blood cultures were only drawn for patients with signs of infection, and the exclusion of infection did not rely on negative blood cultures. According to the above diagnostic criteria, patients with concurrent infections on Days 0–1 were also excluded.

Activities of daily living (ADL) and the Charlson comorbidity index (CCI) were also included to assess the baseline physical condition of patients. ADL refers to fundamental self‐care tasks essential for maintaining independence and is evaluated by the Barthel Index [28]. It is mainly used to measure the functional level of the subjects in basic life skills such as eating, dressing, and moving, as well as their ability to use tools, which is one of the important indicators for assessing the physical functioning of adults. CCI is a tool for calculating comorbidity scores using a predefined list of medical conditions and is associated with mortality [29].

The primary outcomes were 5‐day treatment failure and 6‐week mortality rates. 5‐day treatment failure was defined as the failure of bleeding control or rebleeding within the initial 5 days of admission [16]. Secondary outcomes included the incidence of infections and 1‐year cumulative mortality rates.

### 2.1. Statistical Analysis

Statistical analysis was conducted using SPSS software. Continuous variables were described as follows: if the data were normally distributed, they were expressed as the mean ± standard deviation (SD); otherwise, they were presented as the median and interquartile range. Categorical variables, including etiology, gender, 5‐day treatment failure, 6‐week mortality, and the incidence of nosocomial infections, were expressed in terms of frequencies and percentages.

For continuous variables, we employed the Student′s *t*‐test or the Mann–Whitney *U* test to assess differences between the two groups. Categorical variables were analyzed using the chi‐squared test or Fisher’s exact test, as appropriate. A *p* value of less than 0.05 was considered to indicate statistical significance. Logistic regression analysis was performed to identify factors associated with 5‐day treatment failure and 6‐week mortality. Variables with *p* values of less than 0.05 in univariable analysis were included in the multivariable analysis. For the analysis of 1‐year cumulative mortality rates, the Cox Proportional Hazards Model was utilized to compare the two groups. Statistical significance was set at the 2‐sided 5% level.

## 3. Results

### 3.1. Baseline

Table 1 displays the characteristics of patients. Chronic viral infection was the predominant cause of cirrhosis, representing 54.85% of the total number of cases. The distribution of the two groups of patients was comparable in terms of sex ratio, age, and etiology. Patients in Group A showed lower levels of aspartate aminotransferase (AST), lower albumin (ALB), a lower ADL score, and higher blood urea nitrogen (BUN) levels. Group A had a higher requirement of transfusion and the use of balloon tamponade.

**TABLE 1:** Baseline.

Variables	All patients (*N* = 237)	Group A (*N* = 117)	Group B (*N* = 120)	*p*
Age	53.5 ± 12.21	53.42 ± 11.70	53.58 ± 12.75	0.92
Sex	146 (61.60%)	76 (64.96%)	70 (58.33%)	0.35
Etiology				**< 0.001**
Virus	130 (54.85%)	64 (54.7%)	66 (55%)	
Others	107 (45.15%)	53 (45.3%)	54 (45%)	
Ascites				0.27
No	160 (67.51%)	83 (70.94%)	77 (64.17%)	
Mild	77 (32.49%)	34 (29.06%)	43 (35.83%)	
PVT	37 (15.61%)	21 (17.95%)	16 (13.33%)	0.37
HCC	1 (0.42%)	0 (0.00%)	1 (0.83%)	1.00
Balloon tamponade	19 (8.02%)	19 (16.24%)	0 (0.00%)	**< 0.001**
HB	88.78 ± 26.24	89.17 ± 26.55	88.39 ± 26.03	0.82
PLT	76 (53.5–115)	73 (52–105.5)	78.5 (58–122)	0.16
WBC	5.55 (4.13–7.95)	5.83 (4.04–8.80)	5.34 (4.17–7.40)	0.14
TB	17.2 (12.6–24.05)	17.1 (12.75–22.95)	17.35 (12.53–25)	0.65
ALB	37.19 ± 4.58	36.05 ± 4.37	38.31 ± 4.51	**< 0.001**
CR	67 (57–78)	65 (55.5–78)	69 (57–78)	0.27
ALT	24 (17–38)	22 (17–32.5)	25.5 (17.25–45.25)	0.07
AST	29 (22–41)	27 (20–36)	32.5 (23.25–45.75)	**< 0.001**
GGT	44 (22–91)	42 (20–66)	46 (24–101.75)	0.08
ALP	76 (59–108.5)	72 (56–101.5)	79.5 (63.25–118.5)	0.05
BUN	7.8 (5.5–10.75)	8.88 ± 3.68	6.59 (5.09–10.3)	**0.01**
PT	13.3 (12.3–14.4)	13.30 ± 1.37	13.3 (12.4–14.6)	0.33
Child score: 6	155 (65.4%)	81 (69.23%)	74 (61.67%)	0.28
MELD	8.93 (7.09–10.07)	8.9 (7.45–10.04)	8.89 (6.90–10.19)	0.58
Hemorrhagic shock	70 (29.54%)	40 (34.19%)	30 (25%)	0.15
Transfusion	97 (40.93%)	59 (50.43%)	38 (31.67%)	**< 0.001**
ADL	45 (35–62.5)	40 (35–50)	64.11 ± 19.01	**< 0.001**
CCI	2 (1–3)	2 (1–3)	2 (1–3)	0.87

*Note:* HCC: hepatocellular carcinoma; HB: hemoglobin; PLT: platelet count; ALB: albumin; CR: creatinine; ALT: alanine aminotransferase; AST: aspartate aminotransferase; GGT: γ‐glutamyl transpeptidase; ALP: alkaline phosphatase. Bold values in Table 1 indicate statistical significance (*p* < 0.05).

Abbreviations: ADL, activities of daily living; BUN, blood urea nitrogen; CCI, Charlson comorbidity index; PT, prothrombin time; PVT, portal vein thrombosis; TB, total bilirubin; WBC, white blood cell.

### 3.2. Treatment

**FIGURE 1:**
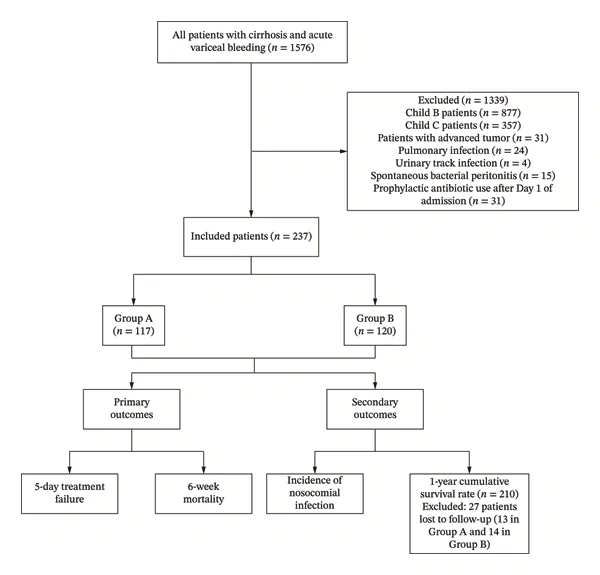
Screening and grouping of patients.

Figure 1 shows the selection process for research objects. All patients with AVB were treated in accordance with the Baveno guidelines immediately after admission. There was no difference between Groups A and B, including vasoactive drugs, PPIs, blood transfusions when necessary, and so on. The specific information on drug treatment and endoscopic/interventional treatment is shown in Table 2.

**TABLE 2:** The treatment of patients with variceal bleeding.

Treatment methods	All patients (*N* = 237)	Group A (*N* = 117)	Group B (*N* = 120)	*p*
Drugs only	29 (12.24%)	10 (8.55%)	19 (15.83%)	
Drugs + endoscopy	107 (45.15%)	54 (46.15%)	53 (44.17%)	
Drugs + TIPS	93 (39.24%)	46 (39.32%)	47 (39.17%)	
Drugs + endoscopy + TIPS	8 (3.38%)	7 (5.98%)	1 (0.83%)	
				0.06

There was also no difference between the two groups in terms of the proportion of PPIs, vasoactive drugs, NSBB drugs, and so on. Endoscopic variceal ligation (EVL) and endoscopic variceal histoacryl injection therapy (EVHT) are the standard treatments in our hospital and the methods of secondary prevention. For patients at high risk of variceal bleeding, transjugular intrahepatic portosystemic shunt (TIPS) is also a recommended treatment method. This strategy did not vary by groups.

**FIGURE 2:**
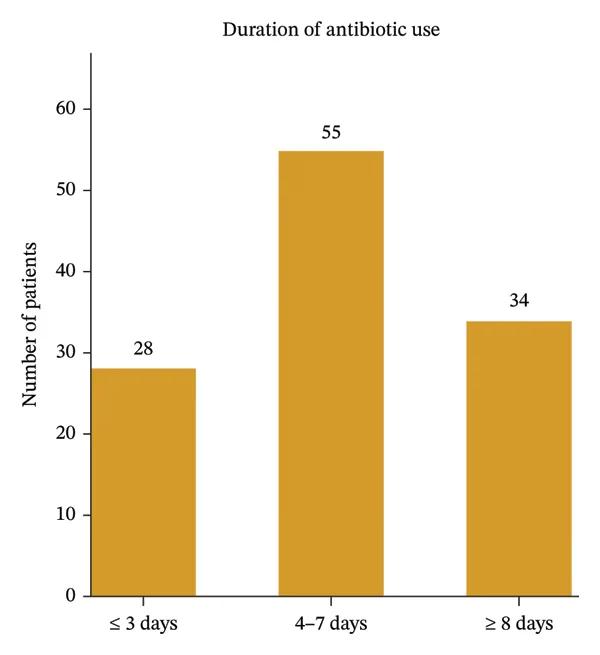
Distribution of antibiotic duration.

For the prevention of infections, the antibiotics most frequently utilized in Group A were cephalosporins (*n* = 89), succeeded by penicillins (*n* = 14) and quinolones (*n* = 14). The duration of antibiotic use varied among patients in Group A. The median duration of antibiotics was 5 days (interquartile range: 4–8 days). A total of 99 patients accepted the administration of prophylactic antibiotics at Day 0; 18 patients started at Day 1. We additionally summarized the distribution of prophylactic antibiotic duration, as shown in Figure 2.

The blood transfusion policy was the same between Group A and Group B. According to the regulations of our hospital, red cell suspension can be transfused when HB is less than 60 g/L. A total of 97 patients received red blood cell transfusions, including 59 in Group A and 38 in Group B. The average transfusion volumes were 3.3 U and 3.5 U, respectively. Plasma transfusion was administered to 15 and 7 patients, respectively, with average volumes of 335 and 370 mL, respectively.

### 3.3. 5‐Day Treatment Failure and 6‐Week Mortality

Five‐day treatment failure occurred in 10 patients in Group A and eight in Group B. No significant difference was found between the two groups (8.5% vs. 6.7%, *p* = 0.63). In the case of failed hemostasis, the continued use of drug treatment or blood transfusion simultaneously eventually stopped the bleeding of 10 patients, and 1 patient received endoscopic treatment. But 2 patients died due to severe hemorrhage, 1 patient refused further treatment, and the subsequent management of 4 patients was unknown.

Among all the cases, a total of 2 patients died within 6 weeks, and both were in Group A. No patients in Group B died within 6 weeks, and there was no statistically significant difference in the mortality rate between the two groups (1.71% vs. 0.00%, *p* = 0.24).

In univariable analysis, PT (OR = 1.60, 95% CI 1.14–2.23, *p* = 0.01), balloon tamponade (OR = 10.98, 95% CI 3.61–33.38, *p* < 0.001), and transfusion (OR = 3.15, 95% CI 1.14–8.72, *p* = 0.03) were significantly associated with 5‐day treatment failure in univariable analysis. The multivariable analysis also revealed a significant correlation between PT (OR = 1.63, 95% CI 1.11–2.38, *p* = 0.01), balloon tamponade (OR = 10.55, 95% CI 3.05–36.46, *p* < 0.001) and treatment failure. The results of univariable and multivariable analyses of 5‐day treatment failure are shown in Table 3.

**TABLE 3:** Univariable and multivariable analyses of potential risk factors for 5‐day treatment failure.

Univariable analysis				Multivariable analysis		
Characteristic	Relative hazard	95% CI	*p* value	Relative hazard	95% CI	*p* value
Age	0.99	0.95–1.03	0.53			
Sex	1.68	0.58–4.88	0.34			
Etiology	2.27	0.78–6.58	0.13			
HB	1.00	0.98–1.02	0.87			
PLT	1.00	0.99–1.01	0.63			
WBC	1.07	0.96–1.19	0.20			
PT	1.60	1.14–2.23	0.01	1.63	1.11–2.38	0.01
TB	1.03	0.98–1.09	0.24			
ALT	0.99	0.97–1.02	0.54			
AST	0.99	0.97–1.01	0.37			
ALB	0.94	0.81–1.04	0.22			
GGT	0.99	0.98–1.00	0.19			
ALP	1.00	0.99–1.01	0.37			
BUN	1.05	0.95–1.17	0.31			
CR	1.00	0.97–1.02	0.85			
Group A/B	1.31	0.50–3.44	0.59			
Balloon tamponade	10.98	3.61–33.38	< 0.001	10.55	3.05–36.46	< 0.001
Nosocomial infection	1.98	0.41–9.55	0.39			
Transfusion	3.15	1.14–8.72	0.03	1.39	0.44–4.39	0.58
Ascites	1.36	0.50–3.64	0.55			

The same factors were evaluated in both univariable and multivariable analyses for 6‐week mortality. In multivariable analysis, WBC count (OR = 1.21, 95% CI 1.00–1.47, *p* = 0.05) remained independently linked to an increased risk of 6‐week mortality. The results of univariable and multivariable analyses of 6‐week mortality are shown in Table 4.

**TABLE 4:** Univariable and multivariable analyses of potential risk factors for 6‐week mortality.

Univariable analysis				Multivariable analysis		
Characteristic	Relative hazard	95% CI	*p* value	Relative hazard	95% CI	*p* value
Age	0.96	0.86–1.08	0.52			
Sex	Inf	0.00–Inf	1.00			
Etiology	Inf	0.00–Inf	0.52			
HB	1.03	0.98–1.08	0.28			
PLT	1.00	0.98–1.02	0.88			
WBC	1.29	1.07–1.54	0.01	1.21	1.00–1.47	0.05
PT	1.34	0.53–3.40	0.53			
TB	1.15	1.02–1.29	0.02	1.13	0.98–1.30	0.10
ALT	0.98	0.90–1.07	0.68			
AST	0.98	0.89–1.08	0.67			
ALB	0.94	0.69–1.28	0.70			
GGT	0.96	0.88–1.05	0.38			
ALP	0.98	0.93–1.03	0.48			
BUN	0.93	0.67–1.30	0.66			
CR	1.00	0.93–1.07	0.94			
Group A/B	Inf	0.00–Inf	1.00			
Nosocomial infection	15.79	0.94–265.88	0.06			
Transfusion	Inf	0.00–Inf	1.00			
Ascites	Inf	0.00–Inf	1.00			

### 3.4. The Incidence of Infection

Despite antibiotic prophylaxis, 7 patients in Group A ultimately developed infections, which included 6 cases of pulmonary infection and 1 case of fever after TIPS surgery. In Group B, 8 patients suffered from nosocomial infections. The incidence of nosocomial infection was comparable between the two groups (5.98% for Group A vs. 6.67% for Group B, *p* = 1.00).

### 3.5. 1‐Year Mortality Rates

Out of 237 patients, 27 were excluded from the 1‐year survival analysis due to incorrect information or a refusal to provide information, among whom 13 belonged to Group A and 14 belonged to Group B.

**FIGURE 3:**
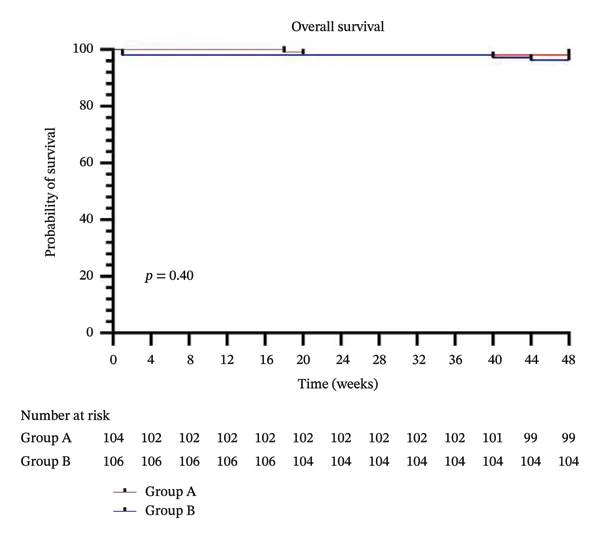
One‐year survival analysis.

The 1‐year cumulative mortality rate was 4.81% for Group A and 1.89% for Group B (*p* = 0.40). Three patients in Group A and two in Group B died between 6 weeks and 1 year after bleeding. The 1‐year survival analysis is shown in Figure 3.

Two patients died within the first 5 days due to severe bleeding. After the first 5 days, information regarding rebleeding was missing for 31 patients. Among the patients with available data, 183 experienced no recurrence of variceal bleeding, whereas 21 patients had at least one episode of rebleeding.

## 4. Discussion

The current study used real‐world data from a major referral center. Our findings indicate that the 5‐day treatment failure and 6‐week mortality are low in patients with Child–Pugh class A cirrhosis and AVB. The 5‐day treatment failure and 6‐week mortality are comparable in patients with or without prophylactic antibiotics.

Cause of bleeding: 24 patients did not undergo endoscopic examination, and the cause of bleeding was uncertain. A total of 129 patients had esophageal varices, 13 patients had gastric varices, and 71 patients had gastroesophageal varices. There were 73 patients with red color signs (21.37% vs. 40%, *p* = 0.002). Due to the lack of data, the Child–Pugh score of 33 patients after bleeding was unknown. Among the other 204 patients, 146 patients’ liver function did not deteriorate, and their Child–Pugh score remained Grade A. Fifty‐four patients’ scores increased compared with those at admission, and Child scores deteriorated to Grade B (26.67% vs. 26.26%). Only 4 patients’ liver functions deteriorated to Grade C (3 in Group A and one in Group B), and there was still no difference in the distribution of the updated Child–Pugh grade (*p* = 0.63). Our findings regarding prophylactic antibiotic use are consistent with Tandon et al.’s study. A total of 381 patients with cirrhosis and AVB were retrospectively included in their study. Among them, only 12% of the cohort was classified as Grade A. Despite prophylactic antibiotics reducing 6‐week mortality from 62% to 35% in patients with Grade C, a survival benefit was not found in Grade A patients.

In their study, the predicted probability of mortality (2.5% vs. 0.4%) was very low in class A patients with or without antibiotic exposure, which was found to be independent of antibiotic use. Similarly, the mortality of Child A patients was 1.71% in Group A and 0% in Group B in our cohort. This is possible because of the overall low mortality in patients with preserved liver function, as well as the improved current treatment strategy.

In another large retrospective analysis, not only patients with AVB but also cirrhotic A/B patients with ulcer bleeding were included. It was confirmed that both the infection rates and mortality rates of Grades A and B patients were relatively lower. Infection and rebleeding within 14 days and 6‐week mortality were not related to the cause of bleeding or antibiotic prophylaxis. Similarly, patients’ baseline was not completely balanced, but even after the PSM analysis of their study, there was still no difference in the outcomes. This study also suggests that the types of antibiotics for prevention may not comply with the guidelines, and the screening for infections may be not comprehensive, with some latent infections possibly being overlooked.

All of the patients in Chang’s study received endoscopic treatment within 12 or 24 h, as recommended by the guidelines [10]. The timing of endoscopy may also have an impact on the prognosis of patients. However, the timing of endoscopic intervention for our patients may not have been strictly managed, which is exactly what we need to reflect on and improve. The optimal timing of endoscopic intervention still requires further research to confirm. But the treatment approaches for bleeding (EVL or EVHT and so on) were not related to the prognosis of patients, both in this study and ours [12].

Two other multicenter retrospective studies conducted in Japan also supported similar conclusions. Although the nosocomial infection rate was slightly different in the study by Wong et al. [13], it was not related to the use of antibiotics and had no impact on patient outcomes. Differently, Ueno’s study included all patients with upper gastrointestinal bleeding, including those with nonvariceal bleeding and other causes. Dewi et al.’s [14] study only included patients with esophageal variceal bleeding who received EVL treatment. Regardless of the cause of the bleeding, the preventive use of antibiotics seemed to be not associated with the outcomes.

In our multivariable analysis, we found balloon tamponade and PT are associated with 5‐day treatment failure. The use of balloon tamponade and elevated PT levels showed that the patient’s bleeding may be more severe. All 2 patients who died within 6 weeks were in Group A. The univariable analysis of 6‐week mortality in our study revealed that WBC counts were associated with 6‐week mortality. But other articles also suggest that nosocomial infections may be an independent predictor of 6‐week mortality and emerge as one of the key prognostic indicators following AVB [30–32], and the prevention of nosocomial infections is considered an important approach to improve patient prognosis and survival [33].

It has been hypothesized that invasive procedures are linked to bacterial infections, as these interventions can facilitate the introduction of pathogens or bacterial translocation [33, 34]. Each invasive procedure, such as tracheal intubation, nasogastric tube, and balloon tamponade, has been shown to independently increase patients’ risk of respiratory infection [33]. Meta‐analysis showed that prophylactic intubation for endoscopy was associated with an increased risk of pneumonia and death in patients with upper gastrointestinal bleeding, especially AVB [35, 36]. This is a warning that patients should be highly selected for intubation because, so far, it has not provided any benefit to patient outcomes.

Consequently, minimizing such procedures may be beneficial to the prognosis of AVB patients. To mitigate the risk of nosocomial infections, it is crucial to strictly adhere to aseptic principles. Additionally, conducting a thorough assessment of patients to identify potential risk factors for infection, such as diabetes and immunosuppression, is crucial. In addition, hepatic encephalopathy (HE) at admission was found to be independently associated with a significantly increased risk of respiratory infection [33], and the prevention of HE may be one of the feasible ways to control infection.

The incidence of nosocomial infection was comparable between the two groups (5.98% vs. 6.67%, *p* = 1.00). A total of 67 patients underwent invasive procedures, including liver biopsy, central venous catheterization, indwelling catheterization, and gastrointestinal decompression. Of these, 32 patients belonged to Group A, and 35 patients were in Group B. But our study did not find a significant association between invasive procedures and infection (*p* = 0.30). It is hypothesized that this may be attributed to the lower infection rate observed in Child–Pugh class A patients. Another study included hospitalized patients with cirrhosis, and endoscopy confirmed upper gastrointestinal bleeding without infection or previous use of antibiotics. It reported a 0% infection rate among Child–Pugh class A patients without antibiotic prophylaxis [37]. That is pretty much the same as we thought and what we found.

This study’s retrospective design inherently introduced certain limitations. The findings from one single center could not represent hospitals in different areas. The baseline characteristics of the two groups were not well‐balanced, and the outcomes might also be related to the preferences of the treating physicians. The following management of cirrhosis and complications of portal hypertension may both be related to the 1‐year prognosis, which should also be taken into account and included in the analysis.

## 5. Conclusion

Neither 5‐day treatment failure nor 6‐week mortality was directly associated with the prophylactic use of antibiotics in patients with Grade A cirrhosis and AVB. Given the favorable clinical outcomes observed in these patients, prophylactic use of antibiotics may not be necessary for patients with Child–Pugh Grade A cirrhosis and AVB.

### 5.1. Lay Summary

For patients with Child A cirrhosis and AVB, prophylactic use of antibiotics is not related to the prognosis of the patients, so prophylactic antibiotic use may be not necessary for them.

## Author Contributions

Guarantors of integrity of entire study, Jiajia He and Xuefeng Luo; study concepts/study design or data acquisition or data analysis/interpretation, all authors; manuscript drafting or manuscript revision for important intellectual content, all authors.

## Funding

The project was supported by National Natural Science Foundation of China (82470640) and the CAMS Innovation Fund for Medical Science (CIFMS) (Grant number 2023‐I2M‐C&T‐B‐085).

## Disclosure

All authors approved the final version of submitted manuscript and agreed to ensure any questions related to the work are appropriately resolved.

## Ethics Statement

All patient data were anonymized to ensure confidentiality, and the study followed the principles outlined in the Declaration of Helsinki and was approved by the Biomedical Research Ethics Committee of West China Hospital, Sichuan University [IRB No. 2022(339)]. Written informed consent was waived because of the retrospective nature of this study.

## Conflicts of Interest

The authors declare no conflicts of interest.

## Data Availability

The data that support the findings of this study are available from the corresponding author upon reasonable request.
